# Manganese and Infant Mortality: Well Water May Raise Death Rates in Bangladesh

**Published:** 2007-07

**Authors:** Julia R. Barrett

Many wells in Bangladesh exceed the WHO threshold for manganese of 0.4 mg/L—in the Araihazar region in eastern Bangladesh, 80% of the wells provide water with manganese concentrations above 0.5 mg/L. A new study now suggests that manganese exposure through drinking water may contribute to Bangladesh’s extremely high infant mortality rate of 54 per 1,000 live births **[*EHP* 115:1107–1112; Hafeman et al.]**.

Well water in Bangladesh already receives close scrutiny for its naturally high levels of arsenic, a known carcinogen. Manganese is also a concern, however, due to research showing associations between exposure and subclinical neurological effects in adults and decreased intellectual function in children. Additionally, neonatal animal studies have linked reduced weight gain and decreased survival to manganese exposure.

An ongoing cohort study in Araihazar, the Health Effects of Arsenic Longitudinal Study (HEALS), provided the framework for investigating whether manganese might affect human infant survival. Of the HEALS participants, 1,628 women met the criteria for the current study: they married before age 40, drank from the same well for most of their reproductive years, and reported at least one live birth. The researchers considered concentrations of manganese and other metals in the wells used by the target population, the mothers’ reproductive history and education, and the families’ socioeconomic status and other factors that can affect infant survival.

Although breastfeeding rates are high in Bangladesh, newborns are often given sugar water in place of colostrum, and most young infants receive complementary foods by age 6 months. Of the 3,824 infants born to the study group, nearly 85% were exposed to water manganese levels above 0.4 mg/L, and 335 died before age 1 year, an elevated risk of death not explained by measured covariates. The finding was more pronounced when restricted to women married after 1991, who appeared to give more complete reproductive histories; among these women, the infant death rate was 82 per 1,000 live births.

No dose–response relationship was seen, though, and the association was not found when analysis was restricted to water samples collected for the current study. The researchers recommend designing a study specifically to investigate the role of manganese exposure in infant mortality, with particular attention to causes of mortality and potential routes of exposure.

## Figures and Tables

**Figure:**
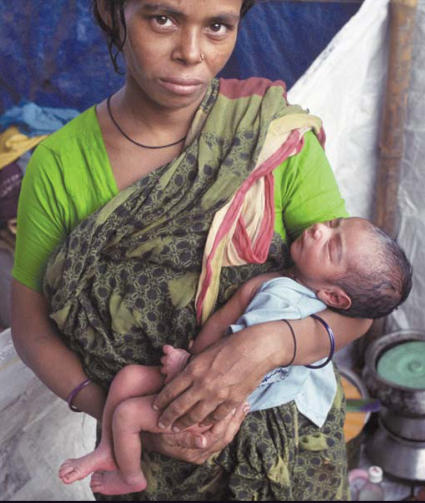
Mother and newborn in Dhaka, Bangladesh

